# Therapy Effects of Wogonin on Ovarian Cancer Cells

**DOI:** 10.1155/2017/9381513

**Published:** 2017-10-18

**Authors:** Jiang Ruibin, Jin Bo, Wan Danying, Zhu Chihong, Feng Jianguo, Gu Linhui

**Affiliations:** ^1^Cancer Research Institute, Zhejiang Cancer Hospital, Hangzhou, Zhejiang 310022, China; ^2^College of Life Science, Zhejiang Chinese Medical University, Hangzhou, Zhejiang 310053, China; ^3^Key Laboratory of Diagnosis and Treatment Technology on Thoracic Oncology, Hangzhou, Zhejiang 310022, China

## Abstract

**Background:**

Wogonin is a plant monoflavonoid and has been reported to induce apoptosis of cancer cells and show inhibitory effect on cancer cell growth. However, the detailed and underlying molecular mechanisms are not elucidated. In this study, we investigated the molecular and biological effects of wogonin in human ovarian A2780 cancer cells.

**Materials and Methods:**

We determined the effects of wogonin on the changes of cell cycling and apoptotic responses of cells. Western blot analysis was used to measure the effects of wogonin on protein expressions.

**Results:**

Our results showed that treatment with wogonin inhibited the cancer cell proliferation, decreased the percentage of G0/G1 subpopulation, and reduced invasiveness of A2780 cells. Exposure to wogonin also resulted in downregulated protein levels of estrogen receptor alpha (ER-*α*), VEGF, Bcl-2, and Akt and increased expressions of Bax and p53. In addition, exposure to wogonin increased caspase-3 cleavage and induced apoptosis in A2780 cells. Our study further showed that MPP, a specific ER-*α* inhibitor, significantly enhanced antitumor effects of wogonin in A2780 cells.

**Conclusion:**

Our results suggest a potential clinical impact of wogonin on management of ovarian cancer.

## 1. Introduction

Ovarian cancer is an estrogen-dependent disease and the leading cause of mortality in women, with more than 204,000 cases diagnosed in the world every year. Eighty-five percent of ovarian cancer is epithelial disease, and surgery is the first-line treatment. Other treatment options such as radiation therapy, hormonal therapy, and chemotherapy may also be applied based on tumor stages [1, 2].

Estrogen stimulation plays vital role in cancer development and progression, which is regulated by estrogen receptor (ER-*α*), for this disease [3–5]. ER-*α* mediates both genomic signaling pathways and nongenomic signaling pathways, regulating cancer cell proliferation [6, 7]. Studies [8–10] have also suggested a potential of ER-*α* overexpression or increase of ER-*α*/*β* expression ratio, on selective growth advantage for ER-*α* positive cells during the development and progression of ovarian cancer. Indeed, exposure to exogenous estrogen was found to promote the viability of ER-positive ovarian cancer cell lines [11, 12]. For example, Choi et al. [13] reported that overexpression of ER-*α*, but not ER-*β*, significantly promoted the growth of ovarian carcinoma A2780 and OVCAR-3 cells, and silencing of ER-*α* expression dramatically reduced cell growth of ovarian cancer BG-1 cells. Thus, targeting estrogen receptor (ER) signaling became a clinical management for ovarian cancer patient. To this setting, tamoxifen, as an agent of selective estrogen receptor modulator (SERM), has been used to treat ovarian stromal tumors. However, tamoxifen may cause severe adverse effects such as increased risk of serious blood clots due to its weak estrogen activity, and the resistance developed during tamoxifen treatment is another clinical challenge [14–16]. Therefore, there is urgent need to develop novel agents targeting and intervening in ER signaling for ovarian cancer treatment with lower toxic and minimized adverse effects to improve clinical outcomes for this disease.

Flavonoids are a class of plant secondary metabolites and polyphenolic compounds that can be taken from natural products [17]. Flavonoids were found to be able to act as antitumor and antioxidant agents [18, 19]. Of them, wogonin from traditional Chinese herb* Scutellaria baicalensis Georgi* has been widely used and researched for allergic and inflammatory diseases with its medication effects of potential “cleansing heat” and “removing toxins” [20, 21]. Previous studies have shown inhibitory effects of wogonin on a number of different cancerous cells [22, 23]. The mechanisms of its anticancer activities include modulation of p53 signaling pathway [24, 25], inducing G1 phase arrest [26], antitumor angiogenesis by inhibition of VEGF [27, 28], and inhibition of apoptosis through the mitochondrial pathway [29]. In addition, a study also demonstrated the antiproliferative activities of wogonin in epithelial ovarian carcinoma (EOC) cells and six primary cultured EOC with disease stages III-IV, and the observed inhibitions on cell growth were reported through phytochemicals-induced cell-cycle modulation and apoptosis induction [1]. These studies indicate that wogonin is a new anticancer agent with enhancement of the curative effect on chemoinsensitive tumors that may clinically benefit ovarian cancer patients.

In this study, we present data showing the detailed mechanisms by which wogonin modulate the ER-*α* signaling pathway and inhibit cancer cell growth of ovarian carcinoma.

## 2. Materials and Methods

### 2.1. Reagents

**Figure 1:**
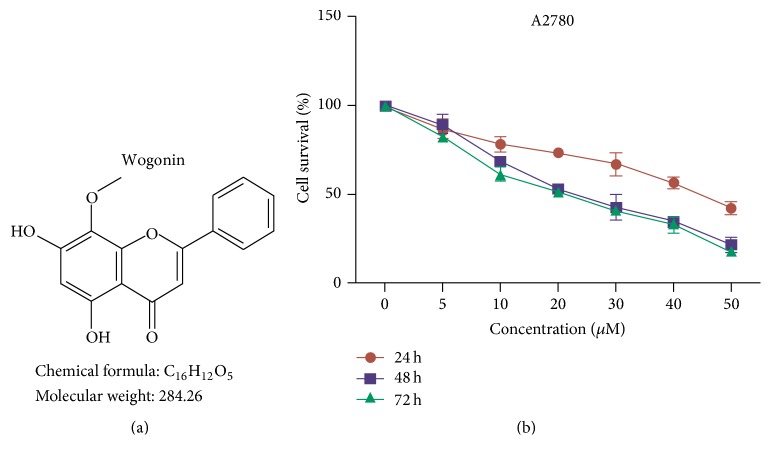
Wogonin inhibits cancer cell growth of A2780 ovarian cancer cells. (a) The chemical structure of the active form of 5,7-dihydroxy-8-methoxyflavone (named as wogonin). (b) Graphs showing the inhibitory effects of wogonin on proliferation of A2780 cells. Data present average results from three independent experiment; SD means the standard deviation (*n* = 3).

A2780 cells were purchased from Sigma-Aldrich Co. (St Louis, MO) and were cultured in RPMI-1640 medium supplemented with 10% fetal bovine serum (FBS, Gibco). Cells were tested with a Cell Culture Contamination Detection Kit (Thermo Fisher Scientific) and results appeared negative for mycoplasma contamination. Wogonin, with a chemical structure shown in Figure 1(a), was purchased from Aokebio (Beijing, China). Methylpiperidinopyrazole (MPP) was purchased from Apexbio. The antibodies were from Abcam (Akt, *β*-actin), Proteintech (caspase-3, cleaved-caspase-3, cyclin D1, CDK4, and CDK6), Santa Cruz Biotechnology (ER-*α*), and Cell Signaling Technology (Bcl-2, Bax, VEGF, and p53).

### 2.2. Cell Viability Analysis

Cells were plated in 96-well plates (5 × 10^3^ cells/well). 24 hours later, cells were exposed to increasing concentrations of wogonin and/or MPP, and DMSO was included as control. MTT assay was performed for measuring cell viability according to manufacturer's instruction (Promega, USA).

### 2.3. Invasion Assay

After treatment with wogonin or MPP for 48 hours, 5 × 10^4^ cells were seeded in cell culture inserts with 1% FBS. Culture medium with 10% FBS was placed outside the chambers. Cells that invaded the attractant of 10% FBS medium were visualized and counted after 48 h. The method was previously reported [30].

### 2.4. Clonogenic Assay

Cells were plated in 60 mm dishes (1000/dish) and were treated with different concentrations of wogonin or MPP for 48 hours. Cells were cultured for two additional weeks. Colonies (>50 cells) were stained with crystal violet prior to being counted under an inverted microscope [30].

### 2.5. Wound Scratch Assay

Cultured cells in confluent monolayer were wounded using a needle to scratch the surface. Cells were then exposed to wogonin for 48 h. Cell movement and initial wounding were imaged and analyzed by Image J software.

### 2.6. Apoptosis Assay

Log-phase growing cells were treated with wogonin for 48 hours. The cells were collected and washed with PBS and were then incubated with staining buffer containing Annexin V-FITC and PI according to manufacturer's instruction (BD Pharmingen, number 556547); apoptotic cells (FITC+/PI−) were quantified by flow cytometry.

For fluorescence microscopy analysis, cells were seeded in 96-well plates at 5 × 10^3^/well and were then exposed to different doses of wogonin and MPP for 48 hours. After treatment, 5 *μ*g/ml Hoechst 33342 and 10 *μ*g/ml PI were added to the cultured cells. 40 minutes later, cells were collected, and fluorescence microscopy analysis was used for assessment of cell morphology.

### 2.7. Cell-Cycle Analysis

Cells were plated in 6-well plates at 2.0 × 10^5^/well. 24 hours later, cells were treated with different concentrations of wogonin for 48 hours. Cells were then collected and fixed with 70% ethanol (prepared by PBS) at −4°C. The fixed cells were incubated with RNase A (100 *μ*g/mL) and PI (50 *μ*g/mL) in PBS for 30 min. The cell cycling was determined using flow cytometry analysis with CXP software, and results were analyzed with Cytomics™ FC 500 software (Beckman).

### 2.8. Western Blot Analysis

A2780 cells were collected and lysed with RIPA lysis buffer (Beyotime, China) containing 1 mM PMSF. 25 *μ*g of cell lysate was loaded onto 10% SDS-polyacrylamide gels for immunoblot analysis. All primary antibodies were used at 1 : 500 to 1 : 1000 dilutions in this study, and beta-actin was included for equal protein loading [31].

### 2.9. Statistical Analysis

Statistical analyses were performed by Student's *t*-test with one-way ANOVA (*P* < 0.05) [32].

## 3. Results

### 3.1. Antitumor Effects of Wogonin on A2780 Ovarian Cancer Cells

We first examined effects of wogonin on cell proliferation of A2780 cells. In this experiment, we used DMSO as control. Our results showed that wogonin dramatically inhibited cell proliferation of A2780 cells, and the inhibitory effect of wogonin on A2780 cells is dependent on the dose of wogonin (Figure 1(b)).

### 3.2. Wogonin Decreased Invasiveness and Migration of A2780 Cells

**Figure 2:**
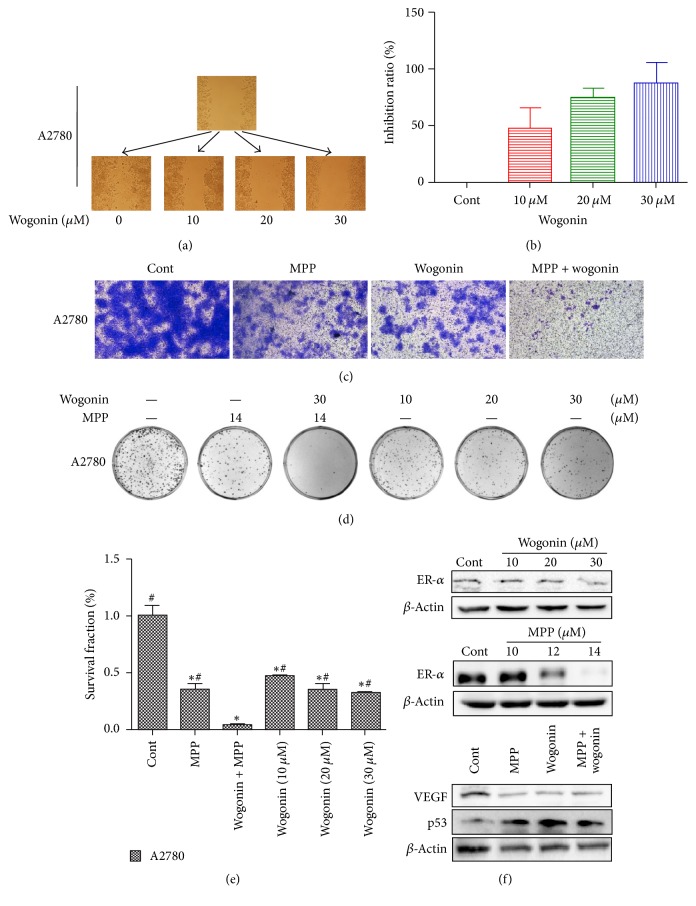
Combination effects of wogonin and MPP on cancer cell invasion and clonogenic survival in A2780 ovarian cancer cells. ((a) and (b)) Wogonin inhibits A2780 ovarian cancer cells migration. Representative images of wound scratch assay showing the effect of wogonin on cell migration. Graph showing the change of inhibition ratio for wound scratch rehealing. (c) Invasion assay. Representative images showing the effect of wogonin on cancer cell invasiveness. (d) Combined effects of wogonin and MPP on clonogenic survival of A2780 ovarian cancer cells. Representative images showing the surviving colonies. (e) Graphs showing the changes of clonogenic survival fraction. The error bars represent the standard error, ^*∗*^*P* < 0.05 versus control group and ^#^*P* < 0.05 versus wogonin + MPP group, *n* = 3. (f) Western blot analysis showing the effects of wogonin and MPP on ER-*α*, VEGF, and p53 protein expression in A2780 ovarian cancer cells. *β*-Actin was included as a loading control. Data present average results from three independent experiments; SD means the standard deviation (*n* = 3).

We next determined the effects of wogonin on invasiveness and migration of A2780 cells. With invasion assay and wound scratch assay, we found that treatment with wogonin dramatically decreased cancer cell capabilities of invasion and migration, and these inhibitory effects are also dose-dependent (Figures 2(a)–2(c)).

We further tested the potential enhancement of antitumor effects of wogonin in ovarian cancer cells where ER-*α* signaling was blocked by treatment of* methylpiperidinopyrazole* (MPP), and our results showed that MPP significantly enhanced the inhibitory effects of wogonin on invasiveness and clonogenic survival of A2780 cells (Figures 2(c)–2(e)). Of interest, Western blot analysis (Figure 2(f)) showed that wogonin also downregulates ER-*α* expression. These results suggest that ER-*α* signaling pathway might interact with the inhibitory effects of wogonin on cancer cell invasiveness and migration of A2780 cells. In addition, our results further showed that exposure to wogonin increased p53 expression and decreased the protein level of VEGF (Figure 2(f)).

### 3.3. Wogonin Induces Apoptosis in A2780 Cells

**Figure 3:**
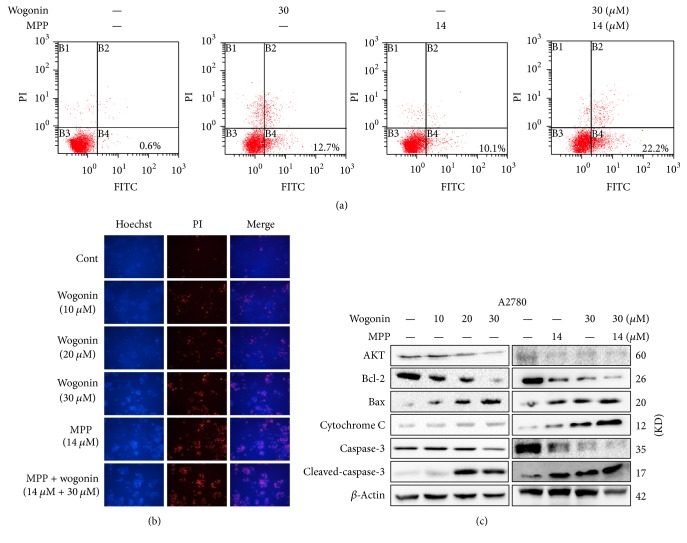
Wogonin treatment induces apoptosis in A2780 ovarian cancer cells. (a) Representative images showing apoptosis detected with Annexin V-FITC and PI staining in A2780 cells. Percentage of the bottom right quadrant and the top right quadrant showing the representative events of early and late apoptosis, respectively. (b) Representative images of fluorescence microscopy analysis showing the effects of treatment with wogonin, MPP, and the combination on nuclei apoptosis and cell necrosis in A2780 cells. Cells were stained with Hoechst and PI, and images were taken under magnification of ×200. (c) The protein expression of Akt, Bcl-2,Bax, cytochrome C, caspase-3, and cleaved-caspase-3. *β*-Actin was included as a loading control.

To determine the potential apoptosis and death as the inhibitory effect of wogonin and MPP in A2780 cells, we performed Annexin V-FITC staining in cells with treatment of wogonin and MPP. Our results (Figure 3(a)) showed that treatment with wogonin or MPP increased both early and late apoptosis in A2780 cells. Of note, the combination of wogonin with MPP resulted in significant increase of the apoptosis-induction effect of wogonin in A2780 cells. Microscopy analyses with fluorescence staining of Hoechst 33342 and PI also demonstrated identical morphological changes of A2780 cells. We detected nuclear modifications of fragmentation and chromatin condensation in cells that were treated with wogonin, and our results suggested these changes are dependent on the dose of wogonin (Figure 3(b)).

The Bcl-2 and caspase cascade play essential roles in balancing antiapoptosis and proapoptosis of cells and integrate a wide range of diverse upstream survival and death signals to determine the fate of cells. In A2780 cells treated with wogonin or MPP, we detected decreased levels of Akt and Bcl-2 (Figure 3(c)). However, exposure to wogonin or MPP increased Bax protein level and caspase-3 cleavage. These results suggest that wogonin and MPP can regulate the expression of Bcl-2 family proteins and the activation of caspase cascade, resulting in increase of apoptosis in ovarian cancer cells.

### 3.4. Wogonin Causes Cell-Cycle Arrest in A2780 Ovarian Cancer Cells

**Figure 4:**
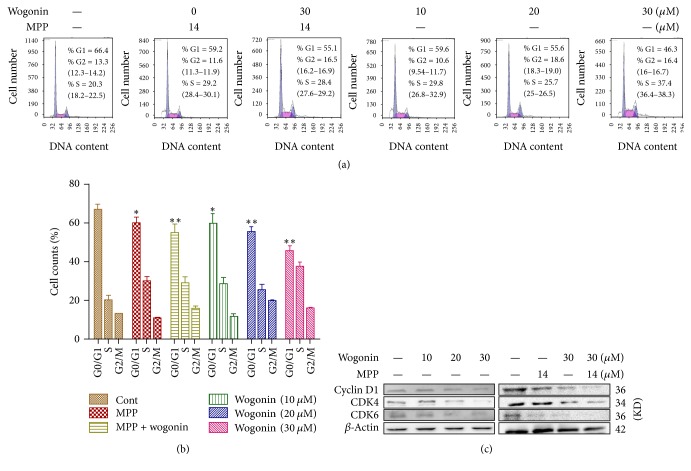
Effects of wogonin on cell cycling distribution of A2780 ovarian cancer cells. (a) Representative results of the flow cytometry analysis with A2780 cells treated with or without wogonin combined with MPP. (b) Graphs showing the changes of the percentage for each cell cycle in A2780 cells. (c) Representative results of Western blot analysis showing the effects of wogonin on the expression of cyclin D1, CDK4, and CDK6. Data present average results from three independent experiments; SD means the standard deviation (*n* = 3). ^*∗*^*P* < 0.05 and ^*∗∗*^*P* < 0.01 versus control group.

Recent studies have shown that wogonin can also induce cell-cycle arrest through the regulation of the expression of cell-cycle regulators in various cancers. In this study, we determined the cell cycling change of A2780 cells when cells were treated with wogonin and MPP. Our results showed that both wogonin and MPP treatments (48 hours) led to dramatic decrease of the G0/G1 subpopulation of A2780 cells (Figures 4(a) and 4(b)). Western blot analysis (Figure 4(c)) further revealed that treatment with wogonin or MPP reduced expressions of G0/G1 phase-related proteins cyclin D1, CDK4, and CDK6. We also found that combined treatment with wogonin and MPP reduced G0/G1 phase and decreased the expressions of cyclin D1, CDK4, and CDK6.

## 4. Discussion

One of the major clinic challenges in treating cancer is to optimize the therapeutic strategy with maximizing therapy efficacy and minimizing adverse effects in patients [33]. To this setting, traditional Chinese medicines (TCM) have been considered as a resource for selection of novel anticancer drugs.

Previous studies have reported that plant flavonoids possess antitumor and anti-inflammatory effects. Studies also revealed that wogonin induces apoptosis through the modulation of apoptotic factors [34, 35] and induces change of cell cycling by regulation of the cyclin D1 expression in human breast cancer [36]. The histopathogenesis of ovarian cancer is very similar to that of breast cancer with the potential of estrogen and its receptors, such as ER-*α*, as carcinogens. However, the correlation of wogonin and ER-*α* expression has not been demonstrated in ovarian cancer. Our present data show that wogonin inhibits cancer cell proliferation and reduces clonogenic survival ability of ovarian cancer cells. Treatment with wogonin also decreases the percentage of G0/G1 subpopulation. Of interest, we observed downregulated expression of ER-*α* in A2780 cells when cells were exposed to wogonin, suggesting that the antitumor activity of wogonin may interact with ER-*α* signaling. In clinical practice, estrogen receptor is recognized as a prognostic factor for breast cancer and a critical reference for clinical management of breast cancer. Although no prognostic value for ER expression has been suggested for ovarian cancers, up to 60% of ovarian epithelial tumors were reported to have ER overexpression, suggesting a potential of regulatory effects of estrogen signaling for deployment and progression of ovarian cancer, and thus targeting ER-*α* signaling may benefit ovarian cancer patients. To this setting, our results suggest wogonin may act on estrogen signaling as a novel therapeutic agent for ovarian tumor treatment.

Several cancer-related proteins, such as p53 and VEGF, play vital roles in cancer development and progression. These proteins are valuable therapeutic targets for cancer treatment [37, 38]. In this study, our results showed that treatments with wogonin, or wogonin combined with MPP, resulted in dramatic increase of p53 protein expression and decrease of VEGF protein expression in A2780 cells. In addition, much effort in screening of TCM as novel therapeutic agents for cancer treatment has been devoted to testing the capability of TCM for apoptosis induction in cancer cells and in xenograft tumors. Apoptosis is a vital phenomenon that controls cancer cell growth and survival. When cancer cells enter apoptosis, mitochondrial cell death and death receptor pathways are two major pathways that are involved in cancer cell programmed death. In mitochondrial cell death signaling, the accumulation of Bcl-2 family proteins and the ratio of Bax/Bcl-2 in mitochondrial membrane determine the potential release of cytochrome C from the intermembrane space of mitochondria to cytoplasm, where it promotes the formation of the apoptosome and activates the caspase cascade that leads to cell apoptosis [39, 40]. However, ER-*α* signaling can protect cells from apoptosis [41]. Of interest, we revealed in this study that wogonin not only modulates expression of Bcl-2 family protein, allowing cell entering apoptosis, but also modulates ER-*α* expression to reduce protection activity of estrogen on apoptosis in ovarian cancer cells. On the other hand, ER-*α* activation regulates expressions of cell-cycle-related proteins, such as cyclin D1 [42]. Cyclin D1 is a regulatory subunit of cyclin-dependent kinases CDK4 and CDK6 and is a key element controlling transition of cells from G1 phase and S phase [43–45]. Of interest, our results also showed that wogonin alone or combined with MPP reduced the protein levels of cyclin D1, CDK4, and CDK6. Taken together, these results also suggest that wogonin can act as an epigenetic therapy agent for ovarian cancer.

In conclusion, the present data suggests a potential clinical impact of wogonin for ovarian cancer patients. However, further investigation of wogonin as an anticancer drug candidate is needed.
